# Pleiotropic Effects of Immune Responses Explain Variation in the Prevalence of Fibroproliferative Diseases

**DOI:** 10.1371/journal.pgen.1005568

**Published:** 2015-11-05

**Authors:** Shirley B. Russell, Joan C. Smith, Minjun Huang, Joel S. Trupin, Scott M. Williams

**Affiliations:** 1 Vanderbilt Genetics Institute, Division of Dermatology, Department of Medicine, Vanderbilt University, Nashville, Tennessee, United States of America; 2 Meharry Medical College, Nashville, Tennessee, United States of America; 3 Department of Genetics, Geisel School of Medicine, Dartmouth College, Hanover, New Hampshire, United States of America; University of Connecticut Health Center, UNITED STATES

## Abstract

Many diseases are differentially distributed among human populations. Differential selection on genetic variants in ancestral environments that coincidentally predispose to disease can be an underlying cause of these unequal prevalence patterns. Selected genes may be pleiotropic, affecting multiple phenotypes and resulting in more than one disease or trait. Patterns of pleiotropy may be helpful in understanding the underlying causes of an array of conditions in a population. For example, several fibroproliferative diseases are more prevalent and severe in populations of sub-Saharan ancestry. We propose that this disparity is due to selection for an enhanced Th2 response that confers resistance to helminthic infections, and concurrently increases susceptibility to fibrosis due to the profibrotic action of Th2 cytokines. Many studies on selection of Th2-related genes for host resistance to helminths have been reported, but the pleiotropic impact of this selection on the distribution of fibrotic disorders has not been explicitly investigated. We discuss the disproportionate occurrence of fibroproliferative diseases in individuals of African ancestry and provide evidence that adaptation of the immune system has shaped the genetic structure of these human populations in ways that alter the distribution of multiple fibroproliferative diseases.

## Prevalence of Fibroproliferative Diseases in Individuals of African Ancestry

Fibroproliferation is a response to tissue injury in which fibroblast-like cells under the influence of immune modulators proliferate and produce extracellular matrix components to heal a wound. Aberrant regulation may result in excessive accumulation of matrix components, i.e., fibrosis. This pathological process, which can occur in multiple tissues, is a common response to injury that leads to scarring and dysfunction of injured tissue and sometimes death [1]. Dermal fibrosis can result in keloids or other types of hypertrophic scars; fibrosis in the kidney, liver, and lung can result in end-stage disease. Fibrosis is also seen in autoimmune diseases, including scleroderma, sarcoidosis, systemic lupus, and in airway remodeling in asthma. Disorders whose pathophysiology is characterized by an exaggerated response to injury are referred to as fibroproliferative diseases. An increased incidence of fibrosis has been widely observed in black populations and has been termed a fibroid [2] or fibroplastic diathesis [3–5]. Fibroid diathesis is used to describe processes in which growth is an essential characteristic, and it depends for the most part on changes caused by inflammation. As a rule, the starting point is tissue injury. “As the element of heredity seems to enter largely into these changes, perhaps the term fibroid diathesis may be admissible as expressing the inherent tendency to this class of changes” [2].

In a series of articles, Anthony P. Polednak suggested that adaptation to the tropical environment in Africa may have involved a tendency toward connective tissue overgrowth as well as hyperpigmentation, and that both tendencies may affect susceptibility to several chronic diseases and response to disease or drug therapies [6–8]. A subset of fibroproliferative disorders that occur at higher frequency and/or with more severe manifestations in people of African ancestry is listed in Table 1. These include keloids [9], glaucoma [10,11], hypertension [12,13], nephrosclerosis [14], scleroderma [15], sarcoidosis [16], uterine fibroma [17], and allergic diseases, including asthma [18–21].

**Table 1:** Relative frequencies of certain fibroproliferative diseases in black and white populations.

Disease	Fold increase in blacks versus whites	References
Asthma^a^	2	[18,77,78]
Glaucoma, primary open-angle^b^	4–5	[10,170]
Hypertension^c^ ^,^ ^d^	1.4–1.6	[8,13,26,171]
Keloids^e^	20	[172]
Left ventricular hypertrophy^d^	2–3	[173,174]
Malignant hypertension^c^	5–7	[29,175]
Nephrosclerosis^c^	3–5	[14, 176–178]
Nephrosclerosis attributed to hypertension^c^	4–20	[179,180] and references cited in [181]
Sarcoidosis^f^	3–17	References cited in [7], and [16, 182–188]
Scleroderma^c^ ^,^ ^e^ ^,^ ^g^	3	References cited in [7], and [189–191]
Systemic lupus erythematosus^f^	2–4	References cited in [7], and [192–195]
Uterine leiomyoma^h^	1.5–3	[17,196–198]

Location of fibrosis

^a^) airway

^b^) eye

^c^) kidney

^d^) cardiovascular

^e^) skin

^f^) lung

^g^) visceral organs

^h^) uterus

Not all fibrotic disorders show increased prevalence in blacks; such inconsistencies are probably due to a variety of etiologies. In the subset of fibrotic disorders listed in Table 1, different organ systems are affected. In keloids, the dermis alone is involved, whereas in scleroderma there is life-threatening fibrosis of skin and visceral organs with a higher prevalence of renal crisis in African Americans [22]; in sarcoidosis any organ system may be involved but severe pulmonary fibrosis is more prevalent in individuals of African descent [23].

There is strong evidence for a genetic role in fibroproliferative disorders, although in most cases they are genetically complex. Genome-wide linkage studies and targeted genome scans have implicated common loci for several fibroproliferative diseases that may have been selected in a similar environment [24]. We [25], and others [7,14,26–29], have suggested that a common etiopathology may operate in these diseases, and common genetic factors may account for their unusual distribution. We propose that the increased prevalence of fibroproliferative diseases in individuals of African ancestry is due to selective pressure for an elevated Th2 response that confers resistance to helminthic infections and concomitantly increases susceptibility to fibrosis.

## Cytokine Profile for Fibrosis

In the late 1980s it was shown, using a panel of mouse CD4^+^ T cells, that two groups of T helper (Th) lymphocytes produced distinct cytokine patterns [30]. Early evidence that Th2 cytokines were profibrotic came from experiments in which administration of IL12, a cytokine that primes Th1 immunity and blocks Th2 immunity, prevented fibrosis in a mouse model of schistosomiasis [31]. The role these cytokines played in the fibrotic response was further supported in studies using mouse models that polarized to either a Th1 or Th2 response to wounding. Using inbred strains of mice that differed in sensitivity to murine leishmaniasis—a Th1-responsive disorder caused by the intracellular trypanosomatid protozoan *Leishmania chagasi*—it was found that polarization of the immune response toward Th1 or Th2 is under genetic control (reviewed in [32,33]). In C57BL/6 mice that are resistant to leishmanial infection, the Th1 cell population expanded and produced IFNγ, whereas susceptible BALB/c mice exhibited a Th2 response characterized by production of IL4. As seen in Table 2, several different exposures induced a fibrotic phenotype in BALB/c but not in C57BL/6 mice.

**Table 2:** Response to exposure by mouse strain.

Mouse strain	Exposure	Cytokine production	Disease phenotype
C57BL/6	Carbon tetrachloride (liver)	Th1 cytokines	Minimal fibrosis
BALB/c	Carbon tetrachloride (liver)	Th2 cytokines	Severe fibrosis [32]
C57BL/6	Nitric oxide synthase blocker	Ratio IFNγ/IL4 = 173	Hypertension with no increase in cardiac collagen
BALB/c	Nitric oxide synthase blocker	Ratio IFNγ/IL4 = 21	Hypertension with increased cardiac collagen and collagen cross-linking [199]
C57BL/6	Angiotensin II	Not reported	No disease
BALB/c	Angiotensin II	Not reported	Dilated cardiomyopathy [200]

In the carbon tetrachloride study, strains of mice that lacked IFNγ defaulted to a Th2 response and developed fibrosis, whereas treatment with anti-IL4 or IFNγ prevented fibrosis, even in BALB/c mice [32]. When the immune response to infection with *Schistosoma mansoni* or to injection with soluble egg antigen was compared between wild type C57BL/6 mice and mice genetically engineered to be deficient in Th1 or Th2 cytokines and/or Th10, Th2 cytokines were profibrotic while Th1 cytokines were inflammatory. Mortality due to either Th1- or Th2-related pathologies was regulated by IL10, which suppressed the production of both type 1 and type 2 cytokines [34]. Gene expression profiling in liver [35] and lung [36] revealed similar results: Th2-polarized mice overexpressed genes involved in fibrogenesis and wound repair, whereas Th1-polarized mice overexpressed genes associated with inflammation-induced tissue damage.

Th2 cytokines also play an important role in human fibrosis [1,37,38]. IL4 and IL13 increase collagen synthesis in human fibroblasts [39–43]; they also promote fibrocyte differentiation from a subset of peripheral blood monocytes. In contrast, the antifibrotic Th1 cytokines IFNγ and IL12 inhibit fibrocyte differentiation [44]. Increased levels of type 2 cytokines have been observed in patients with pulmonary fibrosis [45,46] and hepatic fibrosis [47,48]. Decreased levels of the antifibrotic Th1 cytokines IFNγ and IFNα have been reported for African Americans with keloids compared to those without keloids [49]. While Th2 cytokines were not measured in this study, an increased level of IL6 was observed; IL6 has been shown to promote Th2 differentiation and inhibit Th1 differentiation [50]. An enhanced Th2 response in keloid patients is also supported by: (1) an increased keloid incidence in high school students with allergies [51]; (2) a reduction in collagen synthesis in keloid and scleroderma fibroblasts by Tranilast, a drug developed to control allergies [52,53]; and (3) a correlation of excessive scar formation with IgE levels [54]. Patients with progressive systemic sclerosis also exhibit a predominant type 2 response, which accounts for the endothelial cell injury, fibrosis, and autoantibody production in this disease [55,56]. Mutations in IL13Rα2, a decoy receptor that serves as an off-signal for IL13 [57,58], are associated with systemic sclerosis [59].

## Immunity to Helminths

**Fig 1:**
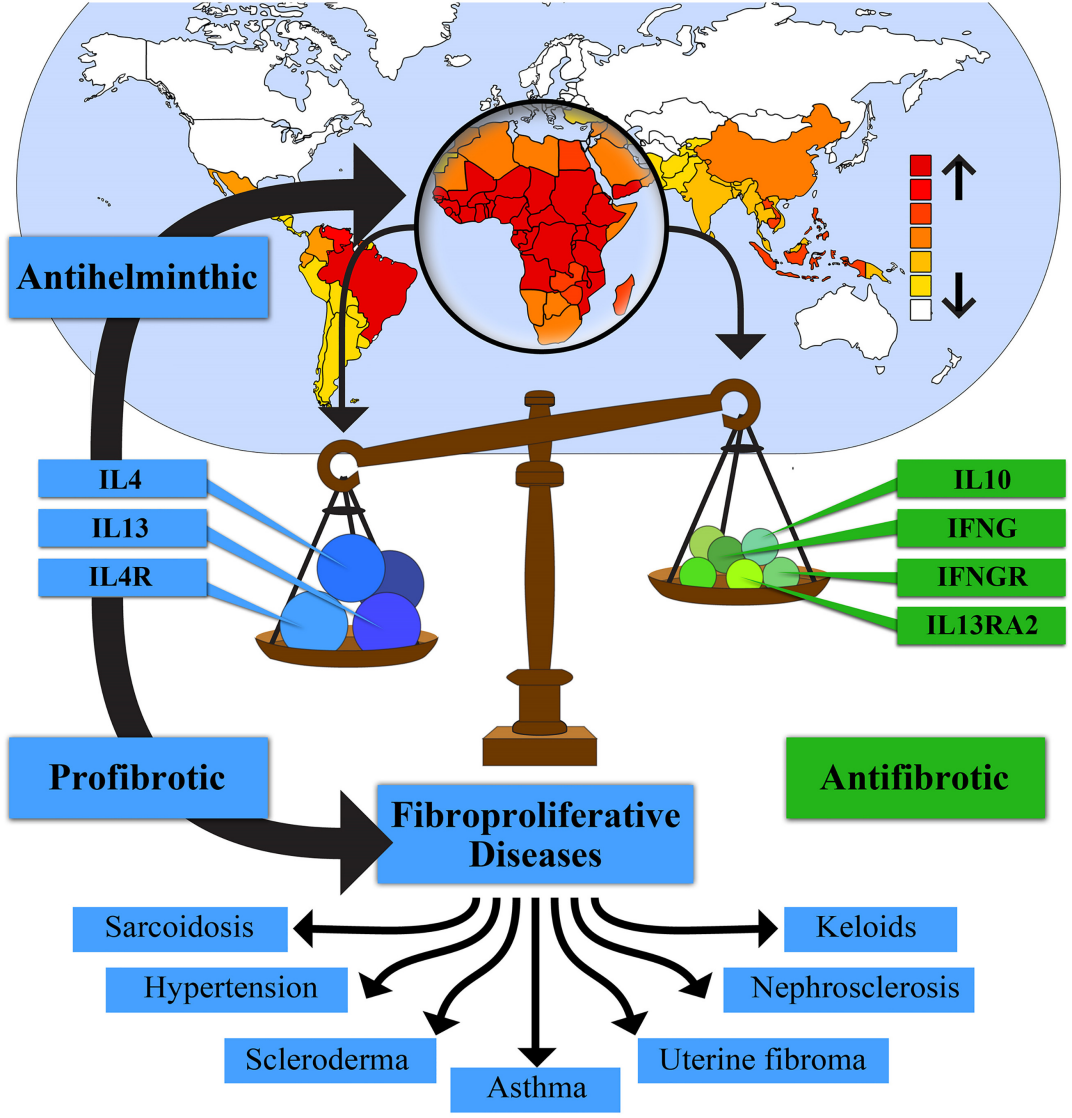
Helminth exposure selects for a protective Th2 immune response that simultaneously increases risk for fibrosis. The high prevalence of helminths in Africa has selected for genotypes favoring an enhanced Th2 immune response characterized by increased levels of interleukin 4 (IL4), interleukin 13 (IL13), and interleukin 4 receptor (IL4R), and other Th2 factors. This selection also decreases Th1 factors, such as interferon gamma (IFNG) and interferon gamma receptor (IFNGR), and Th2 regulatory factors, such as IL10 and interleukin 13 receptor alpha 2 (IL13RA2). These genotypes increase resistance to helminthic infection and contribute to a subset of fibroproliferative diseases that are more common and/or more severe in individuals of African ancestry. Global distribution of helminth species in upper part of figure adapted from Lustigman et al. [168].

Helminths are parasitic worms that cause the most common infectious diseases of humans in developing countries. It is estimated that one billion people in developing areas of sub-Saharan Africa are infected with at least one helminth (Fig 1) [60]. These worms represent a highly diverse group of multicellular eukaryotic parasites, consisting of two phyla, Nematoda, and Platyhelminthes. Infection by any of these parasitic worms, including schistosomes, hookworms, and ascaris, induces a Th2 immune response [60,61]. This response includes the production of cytokines IL4, IL5, and IL13, antibody isotypes IgG1, IgG4, and IgE, and expanded populations of eosinophils, basophils, mast cells, and IL4- and IL13-activated (alternatively activated or M2) macrophages (Fig 1) [61,62]. The adaptive Th2 response mirrors a range of innate helper cell responses that occur upon parasite invasion of epithelium [62]. Interestingly, helminth diversity was observed to correlate with 3,478 gene variants in more than 800 genes in complex networks centered around Th2 cytokines [63]. Th2 immunity is enhanced in environments with a high prevalence of helminthic infection, and may have evolved to isolate and encapsulate the organism and resolve localized extracellular damage [64]. This process can result in tissue injury due to an excess deposition of extracellular matrix, i.e., fibrosis [62,64].

Controlling the Th2 response is imperative to avoid excessive host scarring and other pathological effects. It may also benefit the parasite by damping the immune response, thereby permitting the chronic nature of most helminthic infections. Helminthic infections promote down-regulation of the immune response by expanding populations of immune regulatory cells, including alternatively activated macrophages, Treg cells, and regulatory B cells, by production of IL10 and TGFβ, and by suppression of Th17 [65–68].

The immune response to helminths shares key features with the allergic response. Both are characterized by high levels of IL4, IL5, and IL13, eosinophilia, and abundant IgE production. Several investigators have proposed that a genotype that confers resistance to helminths increases susceptibility to diseases such as allergies, asthma, and autoimmune disease when helminths are not present [69–74]. This is supported by the observation that the geographic distribution of helminth parasitism and allergic disease are complementary [69,75,76], in contrast to the coincident distribution of sickle cell disease and malaria. The prevalence and severity of allergic airway disease are both disproportionately high among African Americans, even after adjustment for demographic and socioeconomic factors [18–21,77,78]. Also consistent are reports of a higher prevalence of atopic dermatitis and elevated IgE levels in individuals of African descent compared with European descent [21,79,80].

During chronic infection, mechanisms that down-regulate both Th1 and Th2 immunity reduce allergic and autoimmune reactions and other pathological effects of the Th2 response, including fibrosis [65–68,81]. Therefore, what we and others argue has been selected historically to deal with parasites may produce an increased fibrotic disease burden in a relatively helminth-free environment where the muted response does not occur. Thus, in the absence of helminthic control mechanisms, individuals genetically predisposed to an increased Th2/Th1 ratio might produce a damaging excess of Th2 cytokines and demonstrate increased incidence and severity of fibroproliferative disease. While direct evidence that helminth infection impacts fibrosis is lacking—save for the complementary distribution of helminth parasitism and asthma (a fibroproliferative disease)—our hypothesis should stimulate research to determine whether this is indeed the case.

Considerable emphasis has been placed on identifying Th2-related genes common to host resistance against helminths and asthma [82–84], but association of such genes with fibrotic disorders that occur with disproportionately high frequency in individuals of African ancestry is lacking. Yet there is support for the notion that Th2 immunity evolved as a rapid repair mechanism in response to extracellular pathogens [64], and that an increased ratio of Th2 to Th1 cytokine responses after injury leads to fibrosis. Moreover, asthma itself is a fibroproliferative disease. As asthma becomes more severe, the airway environment is similar to a chronic wound characterized by secretion of growth factors that induce smooth muscle proliferation, angiogenesis and fibrosis [85,86]. Numerous studies have shown that increased airway wall thickening that includes subepithelial fibrosis results in increased disease severity, including near-fatal and fatal asthma (references cited in [86]).

## Genetics of Resistance to Helminthic Infections

Epidemiological studies in the late 20th century showed that susceptibility to helminthic infection, as defined by worm burden, is a heritable trait with the proportion of variance attributed to genetic effects varying from 0.21–0.44 (reviewed in [70]). Evidence for a genetic predisposition to infection was also provided by ethnic variation in susceptibility, familial aggregation, and individual variation. Differences in susceptibility to helminth infection between ethnic groups was noted early in the 20th century in studies in the southern United States, where a much higher prevalence and intensity of hookworm infection was observed in people of European than African ancestry [87–89]. Additional evidence came from studies that separated effects of relatedness and shared households by statistically analyzing large pedigrees across many households [90].

Identification of specific genes that associate with infection susceptibility provided evidence that immune-related, and especially Th2-related genes, tended to associate with helminth infection. The first genome scans for a parasitic disease associated a major locus that mapped to chromosome region 5q31-q33 with resistance to *S*. *mansoni* infection. This region is rich in Th2 cytokine and Th2 cytokine-related genes [91–93]. A genome study of ascaris infection intensity identified two chromosomal regions, 13q32-q34 and 1p32. *TNFSF13B*, which encodes B lymphocyte stimulator protein and hence is a player in the Th2 response, is in the 13q32-q34 region; this locus also associates with total IgE levels (cited in [70]).

Analysis of *Schistosoma haematobium* infection in Mali revealed that in chromosomal region 5q31-q33, polymorphisms in the *IL13* gene promoter at −1055 and −591 were associated with the infection rate: alleles −1055C and −591A were preferentially transmitted to children with the 10% highest infection rate, whereas −1055T associated with the lowest infection levels [94]. The protective −1055T allele associated with increased *IL13* transcription [95,96] and with resistance to reinfection in a Kenyan cohort [97]. In the latter study, it was also found that the *IL4* −590T allele associated with high IgE, and the *IFNγ* +874T allele associated with high IFNγ production; both increased resistance. Another study in Mali revealed an association between a single-nucleotide polymorphism in the *STAT6* gene at 12q13.3 and intensity of infection by *S*. *haematobium*; this polymorphism had an additive effect with *IL13* −1055 [98].

While most genetic studies of susceptibility to helminth infection have focused on infection intensity, variation in pathology has also been reported, even in the absence of differences in infection intensity. Differences in severity of schistosomal pathology between people of African and European ancestry have been observed in Brazil, with individuals of European ancestry showing increased susceptibility to severe, inflammation-induced pathology [99,100]. A 5-fold increased risk of hepatosplenic disease was observed, despite similar egg counts [101]. Severe hepatosplenism in humans is associated with high levels of Th1 cytokines and low levels of the Th2 cytokine IL5 [102]. Severe disease has been reported more recently to involve elevated levels of the proinflammatory and profibrotic cytokine IL17, produced by Th17 cells [103,104]. However, mutations in the gene encoding the antifibrotic Th1 cytokine IFNγ or its receptor may be important in combating other immunopathological effects caused by Th2 cytokines [105–107]. Individuals with low levels of IFNγ have been reported to be susceptible to severe fibrosis, whereas high levels correlated with reduced fibrosis [107]. Studies in a Sudanese population supported the presence of a major codominant gene controlling hepatic fibrosis in schistosomiasis. Severe hepatic fibrosis due to *S*. *mansoni* infection was also associated with variation at 6q22-23, close to the gene for the IFNγ receptor α chain (*IFNGR1*) [108]. Two polymorphisms (+2109A/G and +3810A/G) in intron 3 of the *IFNγ* gene were associated with periportal fibrosis: the 2109G allele with severe fibrosis, and 3810A with protection from fibrosis. Other studies indicate that 2109G decreases *IFNγ* expression, whereas 3810A increases it [106].

## Evidence for an Immune Adaptation for Resistance to Helminths in Individuals of African Ancestry

As hypothesized by Le Souef et al. [72], “Modern man’s ancestors lived in an environment where infectious tropical diseases would have been endemic.” They postulated that, in this hostile environment, genetic selection for increased Th2 immune responses occurred. In more temperate areas, these pronounced responses would have been less important (and selected against) due to increased mortality from overly vigorous responses to harmless or less common environmental agents. They reviewed evidence that alleles resulting in a heightened Th2 response in several genes, such as *IL4*, *IL4R*, and the *IgE* receptor, are more prevalent in populations with long-term tropical ancestry than in those with long-term residence in temperate regions.

Little has been done to directly determine whether healthy Africans or African Americans exhibit a Th2-biased response. In one small study comparing healthy African children and adults from the Gabonese rain forest to healthy European children and adults from Austria, Wilfing et al. reported an increased frequency of both Th1- and Th2-cytokine-producing T cells in African versus European adults; however, whereas CD4^+^ cells expressing the type 1 cytokines IL2 and IFNγ expanded in both African and European adults, CD4^+^ cells expressing the type 2 cytokines IL4 and IL13 expanded only in African adults [109]. Although these data are consistent with our hypothesis, more extensive studies are needed to determine whether healthy individuals with African ancestry skew toward a Th2 immune response relative to Th1 and Th17 responses.

**Fig 2:**
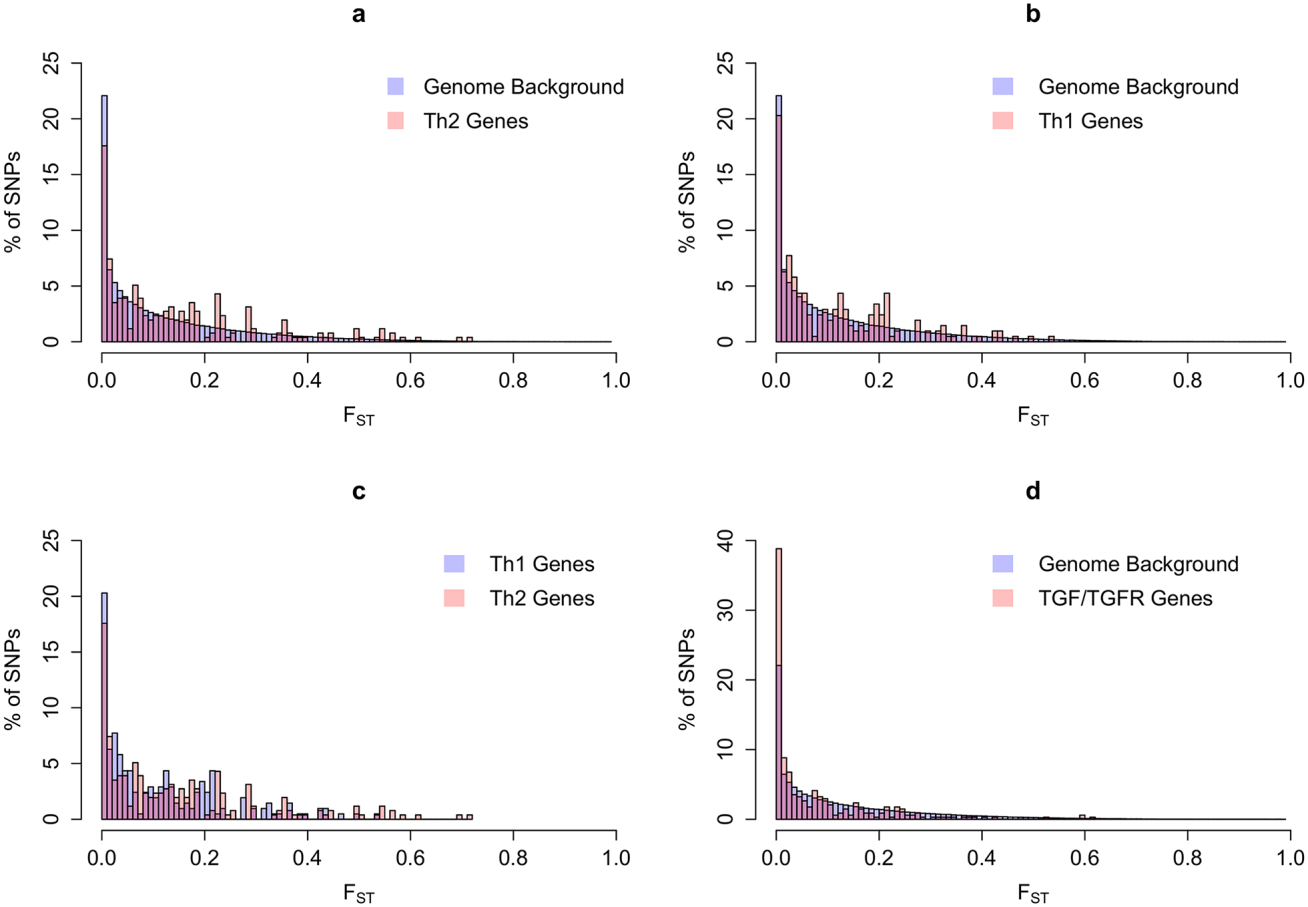
Pattern of differences between YRI and CEU HapMap populations, as determined by Fst between SNPs. (A) Th2 variants compared to background (18 genes and 256 SNPs); (B) Th1 variants compared to background (14 genes and 207 SNPs); (C) A comparison of Fst values for Th2 as compared to Th1 SNPs); (D) TGFβ and TGFβ-receptor variants, as compared to background (6 genes and 340 SNPs). Fst was calculated using the method of Weir and Cockerham [169] and varies from zero (when two populations have identical allele frequencies of a given SNP) to one (when they are fixed for different alleles). Genes used for these analyses are listed in S1 Table.

More recent studies of a subset of genes, and population studies such as 1,000 Genomes [110] and HapMap [111], have supported increased prevalence of alleles associated with resistance to helminths and concomitant susceptibility to fibrosis in West African (YRI), and African American (ASW) populations relative to individuals of Northern European descent (CEU). Of particular interest are polymorphisms in *IL4*, *IL4R*, *IL13*, *IL13RA1*, *IL13RA2*, *IFNG*, *IFNGR1*, and *IL10*, described below and in Table 3, which occur with higher frequency in the recent African descent populations. We also note that allele frequencies in Th2 genes differ more on average between West African and European populations than do differences genome-wide (Fig 2A), supporting the hypothesis that these loci have been exposed to different patterns of selection. Of note, these differences are more pronounced than differences between Th1 genes and the genomic background (Fig 2B and 2C). The frequencies of polymorphisms in East Africa (LWK) seen in 1,000 Genomes are generally similar to YRI. Many of these similarities are also observed in Asian descent populations (S2 Table). Most variants in Th2 and Th1 genes also differ more among these human populations than the genomic background (S1 Fig). However, except for evidence of increased keloid formation in these populations, data on prevalence of fibroproliferative diseases and/or helminthic infection prevalence are too limited to extend our hypothesis beyond Western Africans and African Americans.

**Table 3:** Population-specific allele frequencies (1,000 Genomes, 16 October 2014 release).

rs number	Position	Allele	Prevalence of bolded allele in different populations			Effect of bolded allele
			YRI	CEU	ASW	
*IL4*						
rs2243250	−589	C/**T**	0.833	0.126	0.566	[19,112–114,117,118]^a^ ^,^ ^c^ ^,^ ^d^
rs2070874	−33	**T**/C	0.481	0.126	0.361	[112]^c^ ^,^ ^d^
rs2227284	3017	**T**/G	0.972	0.268	0.779	[112]^c^
rs2243270	intron 2	A/**G**	0.773	0.136	0.549	
rs2243291	intergenic	**C**/G	0.736	0.136	0.508	
rs734244	intron	**T**/C	0.491	0.126	0.402	
*IL4R*						
rs1801275	Q576R	A/**G**	0.852	0.222	0.664	[131,132,135,137,138,140]^d^ ^,^ ^f^
rs1805015	S503 P	T/**C**	0.449	0.167	0.328	[135]^d^
rs1805010	I50V	A**/G**	0.454	0.449	0.467	[133,137,138]^c^
*IL13*						
rs7719175	−7402	**G**/T	0.241	0	0.066	[126]^e^
rs1800925	−1055	C/**T**	0.417	0.177	0.320	[94–97,121,125]^a^ ^,^ ^d^ ^,^ ^e^ ^,^ ^f^
rs2069743	−591	A/**G**	0.292	0	0.123	[94]^e^
rs20541	R110 Q	**A**/G	0.177	0.227	0.189	[120,201,202]^d^ ^,^ ^e^
rs2243204		**T**/C	0.681	0.106	0.467	[125]^f^
*IL13Rα2*						
rs638376		**C**/T	0.994	0.423	0.885	[59]^f^
*TGFβ*						
rs1800470	Pcodon 10L	T/**C**	0.444	0.389	0.377	[13,153,154]^a^ ^,^ ^f^
rs1800469	−509	**A**/G	0.227	0.303	0.205	[153,154,203–205]^a^ ^,^ ^f^
*IL10*						
rs1800896	−1092	**T**/C	0.718	0.480	0.623	[145–147,149]^b^ ^,^ ^c^ ^,^ ^f^ ^,^ ^g^ ^,^ ^h^
rs1800871	−819	**A**/G	0.468	0.207	0.377	[145,146,149]^b^ ^,^ ^c^ ^,^ ^g^ ^,^ ^h^
rs1800872	−592	**T**/G	0.468	0.207	0.377	[145,146,149]^b^ ^,^ ^c^ ^,^ ^g^ ^,^ ^h^
*IFNγ*			YRI	CEU	ASW	
rs2430561	874	**T**/A	0.833	0.576	0.770	[97,160,206]^b^ ^,^ ^f^ ^,^ ^h^ ^,^ ^i^
rs1861494	2109	A/**G**	0.136	0.329	0.156	[106]^f^
*IFNGR1*						
rs1327474	−611	**T**/C	0.972	0.596	0.877	[163]^b^ ^,^ ^h^
*IFNGR2*						
rs9808753	Q64R	A/**G**	0.245	0.141	0.279	[164]^c^

^a^) increased transcription

^b^) decreased transcription

^c^) increased IgE

^d^) allergic disease/asthma

^e^) resistance to helminthic infection

^f^) fibrosis

^g^) increased Th2 cytokines

^h^) forward strand in 1,000 Genomes and HapMap, but apparently earlier literature reporting similar prevalence differences used complementary strand

^i^) sensitivity to helminthic infection

## Interleukin 4

IL4 plays a major role in Th2 differentiation. It induces immature T cells to assume a Th2 phenotype and represses Th1-inducing signals. As a downstream effector, it acts on B cells to produce IgE. Polymorphisms in *IL4* have been associated with increased total serum IgE levels, atopy, and asthma in some populations, but not in others [112–114]. Importantly, *IL4* polymorphisms that associate with asthma severity and elevated IgE in individuals of European descent are more frequent in African Americans than in European Americans (Table 3) [18,19,114,115]. Recent studies have also pointed to a role for private *IL4* mutations in African Americans that may be associated with asthma susceptibility [116]. Somewhat surprisingly, three of the most well-studied of these polymorphisms, at −33, −589, and +3017, associate with asthma and/or elevated IgE in individuals of European descent but not in African populations (Table 3) [19,112–114]; the lack of association at −589T and +3017 in African populations may be due to their high frequency in these populations. Because −589T also associates with expression variation, it provides a putative mechanism to explain the variable patterns of association [117–119]. For example, −589T contributes to lower baseline values of forced expiratory volume (FEV) and higher IgE levels observed in individuals of African descent [21,79,80] and probably the increased prevalence of fibroproliferative disease.

## Interleukin 13

IL13 plays a major role in promoting fibrosis in asthma and schistosomiasis [94,96,97,120,121]. It signals through a common pathway with IL4 [122,123]. Polymorphisms in *IL13* and *IL13Rα1* have been associated with asthma and elevated IgE [57,120,121]. The decoy receptor IL13Rα2 down-regulates IL13 signaling and reduces fibrosis [57,58,124]. As indicated above, −1055T in the *IL13* gene promoter (rs1800925) correlates with different rates of infection in Mali and Kenya [94,97]. This allele, which is associated with lowest infection levels, has also been associated with allergic inflammation, an increased rate of *IL13* transcription [95,121], and systemic sclerosis in individuals of European descent [125]. As seen in Table 3, the T allele is more common in YRI and ASW than in CEU. Also protective against schistosomiasis and occurring at higher frequency in populations of African ancestry is a G allele at a second promoter site (rs771975). These two SNPs are in linkage disequilibrium (LD), making a mechanistic conclusion difficult [126], but haplotype analysis revealed that homozygotes for the doubly protective haplotype TG were less likely to be infected than other subjects. The G allele of a third polymorphism in the *IL13* promoter at −591A/G (−646), rs2069743, was also protective and is present at 33% and 12.3% in YRI and ASW 1,000 Genomes populations, respectively, but is not seen in populations of European descent [94]. A fourth polymorphism, located in the 3’UTR of *IL13*, rs2243204, has been associated with systemic sclerosis in individuals of European ancestry [125]; here too, the risk allele T occurs at higher frequency in African and African-American populations (Table 3). In addition to polymorphisms in *IL13*, a polymorphism in *IL13Rα*2 associated with systemic sclerosis in individuals of European ancestry [59] occurs with higher frequency in individuals of African ancestry (Table 3).

## IL4 Receptor

Both IL4 and IL13 signal through a common pathway by binding to the heterodimeric IL4 receptor (IL4R) composed of the IL4Rα chain and either the common γc chain or the IL13Rα chain. Signaling via IL4Rα plays a critical role in the pathogenesis of asthma [127], with more than a dozen polymorphisms in the gene contributing to asthma risk [128–135]. Several of these susceptibility alleles are more common in African Americans [136–139]. Of particular interest is rs1801275, which causes a glutamine to arginine change at position 576 in the receptor α chain. Arginine at this position is associated with altered IL4 signaling, a shift of the Th1/Th2 balance toward Th2, and susceptibility to asthma and several connective tissue disorders, including systemic lupus and scleroderma [139,140]. The risk allele has a 68% frequency in African Americans but only 20% in populations of European ancestry [137]. Other data demonstrate this allele frequency difference (Table 3) [138]. A nearby variant, rs1805015, which causes a serine to proline change at position 501 associated with atopy [141], is also more prevalent in African Americans, but a third atopy-associated allele (rs1805010) is not (Table 3). While individual alleles are pathogenic, susceptibility is increased by multiple mutations in the *IL4Rα* gene, creating risk haplotypes that are more prevalent in African Americans [138]; these interact with other mutations that influence Th1/Th2 activity, such as those that occur with increased frequency in *IL13* [141].

## Interleukin 10

IL10 is a major immunoregulatory cytokine that downregulates both Th1 and Th2 activity [68,142]. It is effective in preventing fibrosis in several model systems, and suppresses synthesis of procollagen by human scar-derived fibroblasts (cited in [37]). Overexpression of IL10 promotes scarless wound-healing in adult mice [143]. In studies of *S*. *mansoni* infection, severe periportal fibrosis is associated with low concentrations of IL10 and IFNγ [144]. *IL10* promoter haplotypes that include polymorphisms at −1082, −819, and −592 have altered rates of transcription. Alleles causing low *IL10* expression are associated with elevated IgE [145] and increased Th2 cytokines [146]. Allele −1082A is predictive of increased periportal fibrosis in *S*. *mansoni* infection [147]. Population studies have revealed significant differences in the proportion of high or low producer genotypes in populations of European descent versus African Americans [148,149]. A study by Delaney et al. yielded similar results [150]. The combined frequency of low-expression genotypes was significantly higher in African Americans than in European Americans, while the frequency of high expression genotypes in African Americans was less than half that in European Americans. Recently, 1,000 Genomes data confirmed the increased abundance of alleles at promoter sites −1082, −819, and −592 that decrease transcription of IL10 in YRI and ASW compared to CEU (Table 3). These data support a major role for IL10 in preventing fibrosis by down-regulating Th2 cytokines. Thus, the increased frequency of low *IL10* expression genotypes in individuals of African ancestry supports selection for an enhanced Th2 response in this population.

## Transforming Growth Factor β

Increased TGFβ is a component of helminth-mediated down-regulation of the Th2 response [65,71]. While muting the response might be expected to reduce Th2-mediated fibrosis, TGFβ itself promotes a variety of fibrotic conditions [151,152]. Two polymorphisms in *TGF*β1, −509, and +869, which elevate plasma levels of TGFβ1, increase the severity of cystic fibrosis [153] and familial pulmonary hypertension [154]. It has been hypothesized that overexpression of *TGF*β1 contributes to increased morbidity in African Americans [155]. Plasma levels of TGFβ1 in hypertension and end-stage renal disease are higher in African Americans [13,14], and higher levels of TGFβ1 have been reported in normotensive African Americans than in normotensive European Americans. In these studies the +869 variant, encoding a proline, was associated with higher levels of TGFβ1 mRNA and protein, and was initially reported to be more frequent in African Americans [13]. However, increased prevalence was not observed in the 1,000 Genomes database (Table 3). Moreover, the −509 polymorphism associated with increased TGFβ1 is more common in CEU than ASW or YRI (Table 3) [156]. While the TGFβ1 promoter is highly polymorphic, frequencies of different polymorphisms do not differ among racial groups [157]. Several rare variants are present only in individuals with African ancestry but their effects on expression have not been determined [157]. The failure to observe racial differences in frequency of TGFβ polymorphisms and the presence of two common polymorphisms in *TGF*β1 that increase expression but are not more prevalent in individuals with African ancestry suggest that, while *TGF*β1 contributes to fibrosis in multiple populations, increased expression does not directly account for the higher incidence of fibroproliferative diseases in African Americans.

## Interferon γ

Several antifibrotic effects have been attributed to IFNγ, the prototypic Type 1 cytokine. It inhibits fibroblast proliferation and collagen deposition, promotes fibroblast apoptosis, and inhibits the production and profibrotic action of TGFβ (reviewed in [38]). It inhibits development of fibrosis in vivo [158], and reduces the extracellular matrix in animal models of fibrosis [159]. Decreased levels of IFNγ have been observed in the blood of keloid patients [49]. In a Sudanese population infected with *S*. *mansoni*, low levels of IFNγ were associated with severe fibrosis, whereas high levels correlated with reduced fibrosis [107]. However, high levels of IFNγ have been reported to be protective against infection with *S*. *mansoni* [97].

In 1999, Pravica et al. identified a polymorphism in intron 1 at position +874 of *IFN*γ, and found that the A allele at this position correlated with a higher copy number of CA repeats that altered an NFkβ binding site, resulting in decreased IFNγ production [160]. Several studies reported that African Americans have a higher frequency of the allele that decreases production than do European Americans (Table 3) [148,150,161,162]. In Delaney et al. [150], the risk allele frequency was 0.804 in an African American population and 0.61 in a European American population, and was also higher in YRI and ASW (Table 3). As described previously, two polymorphisms, +2109A/G (rs1861494) and +3810A/G, in intron 3 of the *IFNγ* gene associated with periportal fibrosis in a Sudanese population: the 2109G allele decreased *IFNγ* expression and correlated with severe fibrosis, whereas 3810A increased *IFNγ* expression and protected from fibrosis [106]. Somewhat surprisingly, the 2109G allele that associated with low IFNγ production was more frequent in CEU (0.33) than in YRI (0.14) or ASW (0.16) (Table 3). The effect of these alleles on resistance to infection has not been reported; however, high levels of IFNγ have been associated with resistance to infection, suggesting that the low prevalence of the 2109G fibrosis risk allele in African populations may be protective against infection [97].

## IFNγ Receptor (IFNGR)

IFNGR is a heterodimer consisting of two chains—IFNGR1 encoded on chromosome 6 and IFNGR2 on chromosome 21. Mutations in *IFNGR1* have been reported to affect fibrosis. Studies in a Sudanese population indicated that a major codominant locus controlling hepatic fibrosis in schistosomiasis was at 6q22-q23, close to the gene for the IFNGR1 chain [108]. Six polymorphisms in the *IFNGR1* promoter region associated with pulmonary mycobacterial disease [163]. Two (−611 and −56) were polymorphic in all study populations, which included African Americans, Europeans, and Koreans. The −611 polymorphism associated with decreased *IFNGR1* expression was approximately 1.5 times more common in African Americans than in European Americans. Similar differences in frequency among YRI, ASW, and CEU were seen in 1,000 Genomes (Table 3). A polymorphic allele in *IFNGR2* associated with increased IgE was also more common in Africans and African Americans than in Europeans (Table 3) [164].

## Differentiation between African and European Populations with Respect to Th2, Th1 and TGFβ Genes

As documented above, selection has enhanced Th2 gene expression in populations from sub-Saharan Africa that, we argue, is in response to a greater burden of helminthic infections. As a further consequence of this selection, we predict that Th2 alleles will vary more between sub-Saharan populations and European populations than the genomic background. We tested this assumption using the metric, Fst, which represents a measure of genetic differentiation (Fig 2). Consistent with our hypothesis, Th2 gene SNPs represent an enrichment of variants that differ more between sub-Saharan and European populations than does the genetic background (Fig 2A). Differentiation among Th1 gene SNPs appears to follow more closely the genome-wide patterns (Fig 2B and 2C). This comparison includes all SNPs in Th1 and Th2 genes without considering their function. In contrast to the findings for Th2 and to a lesser extent for Th1 genes, differentiation of TGFβ genes and their receptors between populations appears to be identical to the genomic background (Fig 2D).

In summary, individuals of African ancestry have an increased incidence of fibrotic disorders. While the distribution of these diseases supports a genetic contribution, the disorders themselves do not confer a selective advantage. We argue that this pattern of variation can be explained by selection for resistance to diseases caused by helminthic worms prevalent in sub-Saharan Africa. Resistance is achieved by a shift in the immune system toward an enhanced Th2 response to injury that coincidentally results in fibrosis. The exaggerated response to tissue injury resulting in fibrosis can be accounted for in large part by genes that cause increased expression or functionality of Th2 cytokines and their receptors, decreased expression of IL10, and decreased activity of antifibrotic cytokines and receptors of the Th1 system. The allelic distribution of variants that affect the Th2 response are highly concordant with this hypothesis; there is greater differentiation in variants in Th2-related genes compared to the genetic background between African and non-African populations, an observation consistent with selection favoring Th2 enrichment in Africa. While the fibrotic side effects of an increased Th2/Th1 cytokine ratio are a serious consequence, in regions where helminths are common, these side effects are less dangerous than the diseases they protect against. Moreover, chronic helminth infections mute the Th2 response, limiting the fibrotic side effects. Recent migration out of Africa to regions with fewer helminths has, therefore, resulted in a less regulated Th2 pattern, in which frequency and severity of many Th2-related diseases are increased in African Americans.

We hypothesize that genotypes that favor a skewed Th2 response in individuals of African ancestry account for the pattern of multiple fibroproliferative diseases. The increased prevalence of Th2 genotypes that increase resistance to helminthic infection and the increased frequency and severity of a subset of fibrotic disorders in individuals of African ancestry are consistent with a skewed Th2 response; however, more research is needed to determine whether healthy Africans and African Americans exhibit a skewed Th2 response.

Other immunologic differences selected in response to bacterial, viral, and other parasitic challenges in Africa also exist. For example, differences in response to evoked inflammation [165], TLR signaling [166], and stimulation of Th17 expression [167] have been reported which may contribute to increased inflammation and/or fibrosis in African Americans. Our hypothesis is not meant to explain all prevalence differences seen even in the disorders listed in Table 1. However, the high likelihood that helminthic selection for an enhanced Th2 profile contributes to all of the diseases mentioned is based, to a large extent, on the global distribution of helminths, as shown in Fig 1, and the strong evidence for an ancestral genotype that promotes high Th2 expression, decreased IL10 expression, and variably decreased Th1 expression in individuals of African ancestry. This hypothesis, which is testable, is a reasonable starting point for further study.

## Supporting Information

S1 Fig Pattern of differences between HapMap populations as determined by FST between SNPs in Th1 and Th2 genes. A) Th2 variants compared to background (18 genes and 256 SNPs) between CEU and LWK; B) Th1 variants compared to background (14 genes and 207 SNPs) between CEU and LWK; C) Th2 variants compared to background (18 genes and 256 SNPs) between CEU and JPT; D) Th1 variants compared to background (14 genes and 207 SNPs) between CEU and JPT. CEU—Northern and Western European, Utah; LWK—Luhya, Kenya; JPT—Japanese, Tokyo
(DOCX)

S1 Table Genes used in F_st_ analyses. (DOCX)

S2 Table Population allele frequencies (from 1,000 Genomes 16 October 2014 release). (DOCX)
