# Supplementary material for: Pleiotropic Effects of Immune Responses Explain Variation in the Prevalence of Fibroproliferative Diseases
Source: PLoS Genet. 2015 Nov 5;11(11):e1005568. doi: 10.1371/journal.pgen.1005568 (PMC4634921; doi:10.1371/journal.pgen.1005568)

S1 Figure. Pattern of differences between HapMap populations as determined by F_ST_ between SNPs in Th1 and Th2 genes. A) Th2 variants compared to background (18 genes and 256 SNPs) between CEU and LWK; B) Th1 variants compared to background (14 genes and 207 SNPs) between CEU and LWK; C) Th2 variants compared to background (18 genes and 256 SNPs) between CEU and JPT; D) Th1 variants compared to background (14 genes and 207 SNPs) between CEU and JPT. CEU – Northern and Western European, Utah; LWK – Luhya, Kenya; JPT – Japanese, Tokyo

1.
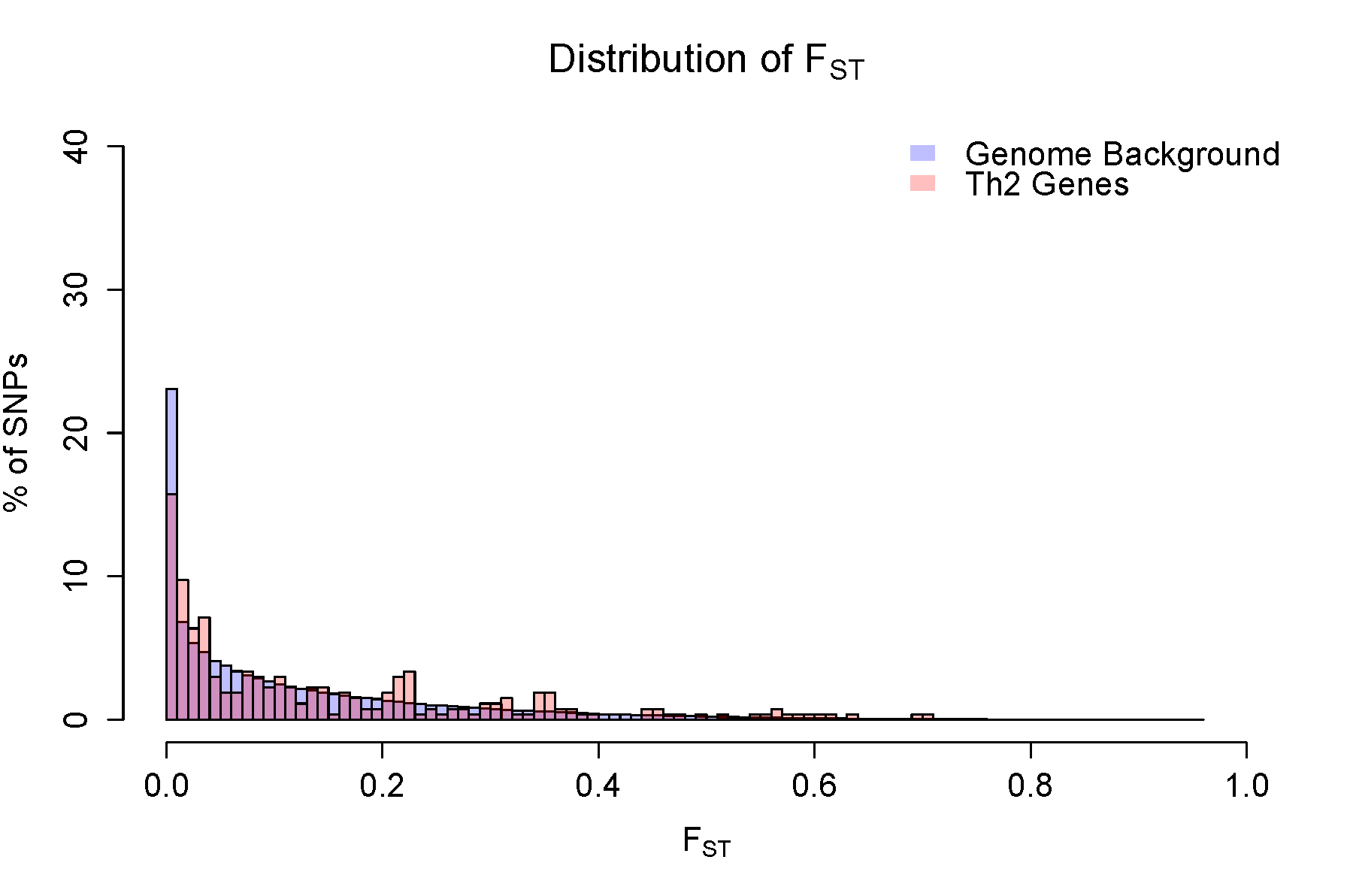

2.
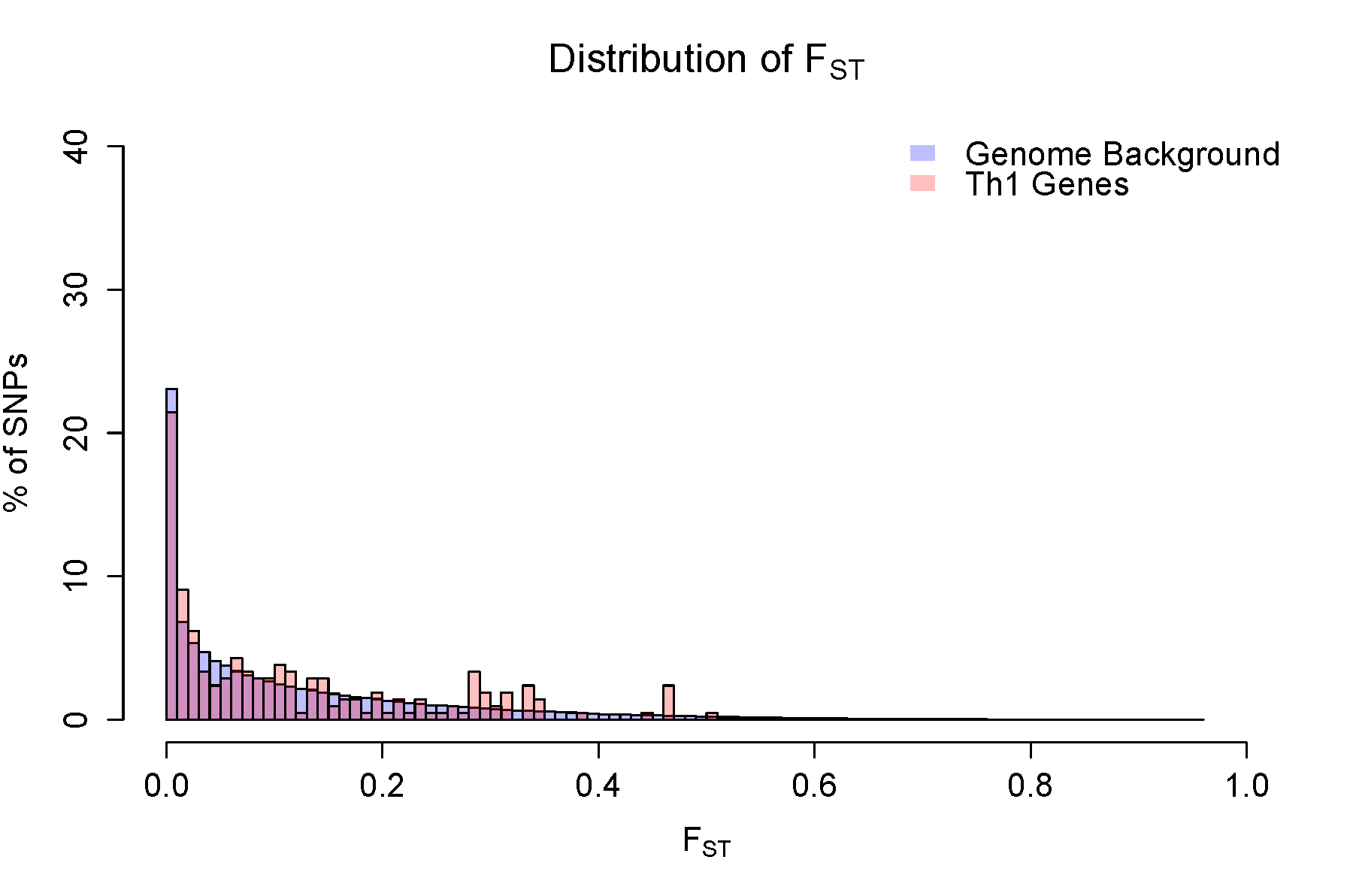

3.
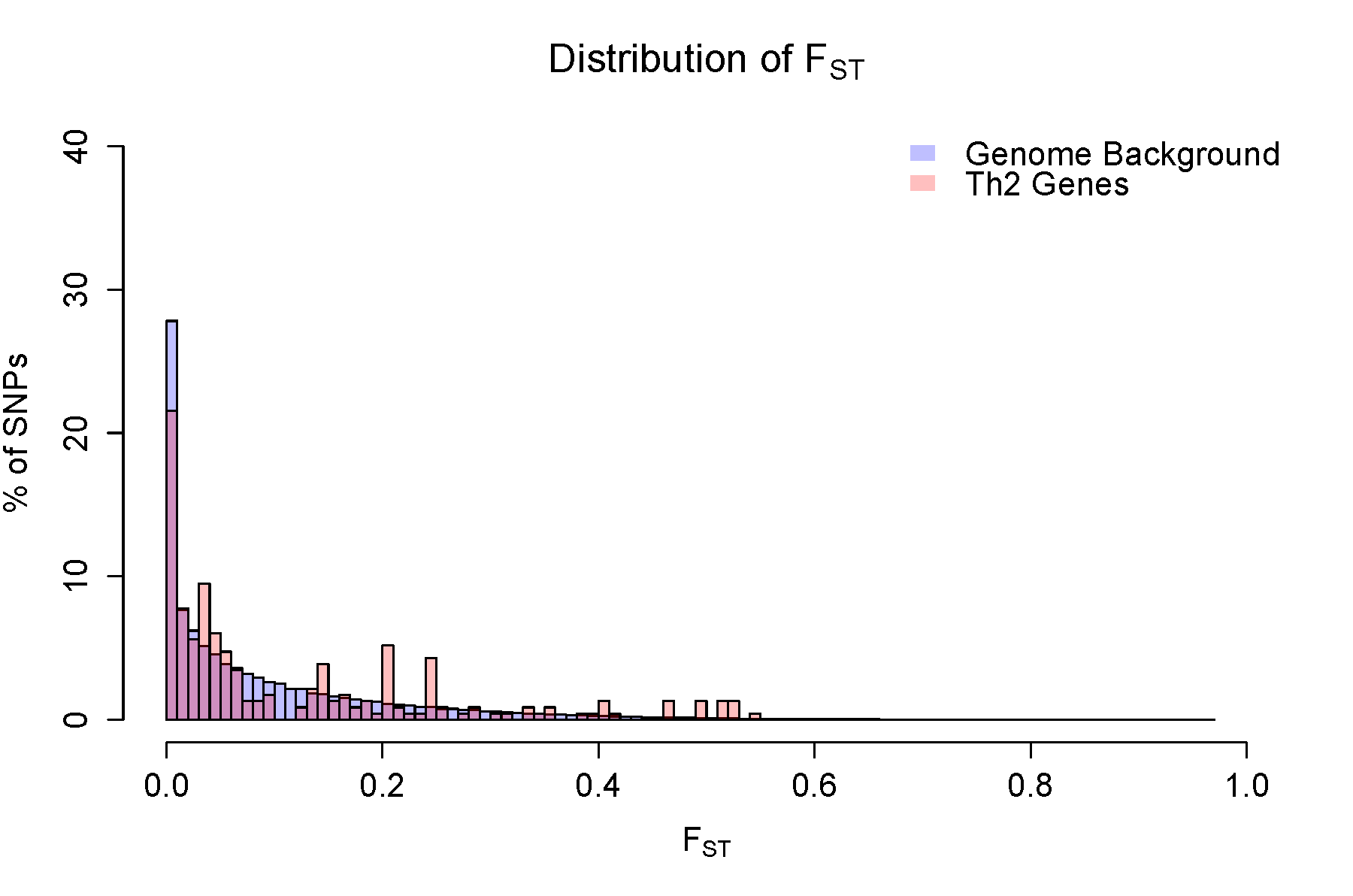

4.
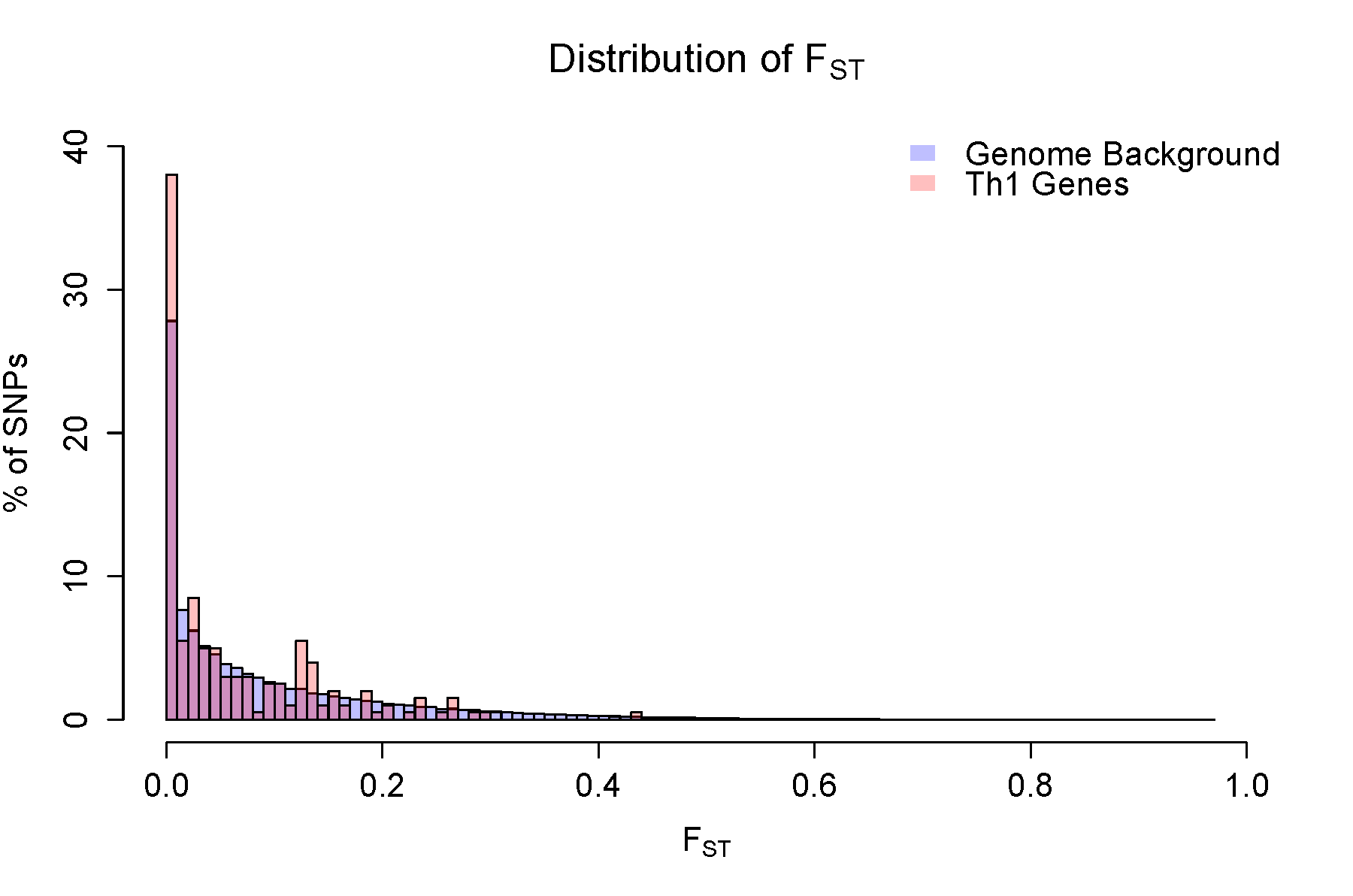

Supplement: S1 Fig — A) Th2 variants compared to background (18 genes and 256 SNPs) between CEU and LWK; B) Th1 variants compared to background (14 genes and 207 SNPs) between CEU and LWK; C) Th2 variants compared to background (18 genes and 256 SNPs) between CEU and JPT; D) Th1 variants compared to background (14 genes and 207 SNPs) between CEU and JPT. CEU—Northern and Western European, Utah; LWK—Luhya, Kenya; JPT—Japanese, Tokyo (DOCX) [file pgen.1005568.s001.docx]
