# Supplementary material for: Pleiotropic Effects of Immune Responses Explain Variation in the Prevalence of Fibroproliferative Diseases
Source: PLoS Genet. 2015 Nov 5;11(11):e1005568. doi: 10.1371/journal.pgen.1005568 (PMC4634921; doi:10.1371/journal.pgen.1005568)
Supplement: S1 Table — (DOCX) [file pgen.1005568.s002.docx]

S1 Table. Genes used in F_st_ analyses

| **Th1 genes** | **Th2 genes** | **TGFβ genes** |
| --- | --- | --- |
| *TNFRSF1B* | *IL10* | *TGFBR3* |
| *IL12RB2* | *IL1RL1* | *TGFB2* |
| *IL12A* | *IL5RA* | *TGFBR2* |
| *IL2* | *IL5RB/CSF2RB* | *TGFBR1* |
| *IL12B* | *IL17RB* | *TGFB3* |
| *LTA* | *IL3* | *TGFB1* |
| *TNF* | *IL5* |  |
| *IFNGR1* | *IL13* |  |
| *IL2RA* | *IL4* |  |
| *TNFRSF1A* | *IL9* |  |
| *IFNG* | *IL33* |  |
| *IL12RB1* | *IL10RA* |  |
| *IFNGR2* | *IL25* |  |
| *IL2RB* | *IL4R* |  |
|  | *IL13RA2* |  |
|  | *IL13RA1* |  |
|  | *IL9R* |  |
