# Supplementary material for: Pleiotropic Effects of Immune Responses Explain Variation in the Prevalence of Fibroproliferative Diseases
Source: PLoS Genet. 2015 Nov 5;11(11):e1005568. doi: 10.1371/journal.pgen.1005568 (PMC4634921; doi:10.1371/journal.pgen.1005568)
Supplement: S2 Table — (DOCX) [file pgen.1005568.s003.docx]

S2 Table. Population Allele Frequencies (from 1000 Genomes 16 October 2014 release) ^1^

| rs number | Position | Allele |  |  |  |  |  |  |
| --- | --- | --- | --- | --- | --- | --- | --- | --- |
|  |  |  | YRI | CEU | ASW | LWK | JPT | CHB |
| *IL4* |  |  |  |  |  |  |  |  |
| rs2243250 | -589 | C/**T** | 0.833 | 0.126 | 0.566 | 0.808 | 0.726 | 0.738 |
| rs2070874 | -33 | **T**/C | 0.481 | 0.126 | 0.361 | 0.581 | 0.726 | 0.738 |
| rs2227284 | 3017 | **T**/G | 0.972 | 0.268 | 0.779 | 0.980 | 0.798 | 0.801 |
| rs2243270 | intron 2 | A/**G** | 0.773 | 0.136 | 0.549 | 0.697 | 0.726 | 0.728 |
| rs2243291 | intergenic | **C**/G | 0.736 | 0.136 | 0.508 | 0.687 | 0.721 | 0.733 |
| rs734244 | intron | **T**/C | 0.491 | 0.126 | 0.402 | 0.581 | 0.726 | 0.728 |
|  |  |  |  |  |  |  |  |  |
| *IL4R* |  |  |  |  |  |  |  |  |
| rs1801275 | Q576R | A/**G** | 0.852 | 0.222 | 0.664 | 0.843 | 0.111 | 0.184 |
| rs1805015 | S503P | T/**C** | 0.449 | 0.167 | 0.328 | 0.510 | 0.077 | 0.092 |
| rs1805010 | I50V | A**/G** | 0.454 | 0.449 | 0.467 | 0.530 | 0.620 | 0.461 |
|  |  |  |  |  |  |  |  |  |
| *IL13* |  |  |  |  |  |  |  |  |
| rs7719175 | -7402 | **G**/T | 0.241 | 0 | 0.066 | 0.076 | 0 | 0 |
| rs1800925 | -1055 | C/**T** | 0.417 | 0.177 | 0.320 | 0.449 | 0.159 | 0.165 |
| rs2069743 | -591 | A/**G** | 0.292 | 0 | 0.123 | 0.172 | 0 | 0 |
| rs20541 | R110Q | **A**/G | 0.177 | 0.227 | 0.189 | 0.242 | 0.298 | 0.320 |
| rs2243204 |  | **T**/C | 0.681 | 0.106 | 0.467 | 0.657 | 0.091 | 0.102 |
|  |  |  |  |  |  |  |  |  |
| *IL13Rα2* |  |  |  |  |  |  |  |  |
| rs638376 |  | **C**/T | 0.994 | 0.423 | 0.885 | 0.968 | 0.112 | 0.138 |
|  |  |  |  |  |  |  |  |  |
| *TGFβ* |  |  |  |  |  |  |  |  |
| rs1800470 | P10L | T/**C** | 0.444 | 0.389 | 0..377 | 0.439 | 0.476 | 0.481 |
| rs1800469 | -509 | **A**/G | 0.227 | 0.303 | 0.205 | 0.207 | 0.471 | 0.476 |
| *IL10* |  |  |  |  |  |  |  |  |
| rs1800896 | -1092 | **T**/C | 0.718 | 0.480 | 0.623 | 0.672 | 0.947 | 0.966 |
| rs1800871 | -819 | **A**/G | 0.468 | 0.207 | 0.377 | 0 .389 | 0.639 | 0.743 |
| rs1800872 | -592 | **T**/G | 0.468 | 0.207 | 0.377 | 0.394 | 0.639 | 0.743 |
|  |  |  |  |  |  |  |  |  |
| *IFNγ* |  |  | YRI | CEU | ASW | LWK | JPT | CHB |
| rs2430561 | 874 | **T**/A | 0.833 | 0.576 | 0.770 | 0.843 | 0.928 | 0.859 |
| rs1861494 | 2109 | A/**G** | 0.136 | 0.329 | 0.156 | 0.101 | 0.529 | 0.359 |
|  |  |  |  |  |  |  |  |  |
| *IFNGR1* |  |  |  |  |  |  |  |  |
| rs1327474 | -611 | **T**/C | 0.972 | 0.596 | 0.877 | 0.975 | 0.976 | 0.927 |
|  |  |  |  |  |  |  |  |  |
| rs9808753 | Q64R | A/**G** | 0.245 | 0.141 | 0.279 | 0.162 | 0.514 | 0.422 |

^1^ YRI – Yoruba, Nigeria; CEU – Northern and Western European, Utah; LWK – Luhya, Kenya; ASW – African-Americans SW USA; JPT – Japanese, Tokyo; CHB- Han Chinese, Beijing
